# Utility of the Caprini risk assessment model in guiding venous thromboembolism prophylaxis after abdominoplasty

**DOI:** 10.1016/j.jpra.2025.09.017

**Published:** 2025-09-17

**Authors:** Abdulaziz Asiry, Anas Sayegh, Dimitri Gangloff, Hatan Mortada, Silvia Gandolfi, Elise Lupon

**Affiliations:** a King Saud Bin Abdulaziz University for Health Sciences, Jeddah, Saudi Arabia; b Department of Surgery, Faculty of Medicine, Jazan University, Jazan, Saudi Arabia; c Plastic and Reconstructive Surgery Department, Rangueil University Hospital Center of Toulouse; d Division of Plastic Surgery, Department of Surgery, King Saud University Medical City, King Saud Univesity, Riyadh, Saudi Arabia; e Institut Universitaire Locomoteur et du Sport, Pasteur 2 Hospital, University Côte d’Azur, Nice, France; f Laboratory of Molecular PhysioMedicine (LP2M), UMR 7370, CNRS, University Côte d'Azur, Nice, France

**Keywords:** Abdominoplasty, Venous thromboembolism, Caprini risk assessment model, Low molecular weight heparin, Body contouring

## Abstract

**Introduction:**

Abdominoplasty carries a significant risk of venous thromboembolism (VTE). Two main strategies have been described for prophylaxis: systematic administration of low molecular weight heparin (LMWH) and risk-stratified management using the Caprini score. This study aimed to assess thromboembolic risk in abdominoplasty patients and compare the cost-effectiveness of a Caprini-based strategy with routine prophylaxis.

**Methods:**

A retrospective review was performed on 219 patients who underwent abdominoplasty at a university plastic surgery unit where systematic LMWH prophylaxis was standard. Demographic data, Caprini score components, complications, and costs were analyzed, and a cost-effectiveness comparison between systematic LMWH and a Caprini-based approach was performed.

**Results:**

The mean Caprini score was 3.3 ± 1.1 (range 1–10). All patients received LMWH for a mean of 14.9 ± 1.5 days. One patient (0.45 %) developed pulmonary embolism, and 13 (5.9 %) developed hematomas. Cost analysis indicated that a risk-stratified approach could reduce LMWH expenditure by 44.5 %.

**Conclusions:**

Applying the Caprini score for VTE prophylaxis in abdominoplasty may reduce unnecessary anticoagulation and associated complications, while generating substantial cost savings for centers that do not currently use this model.

## Introduction

Surgical procedures, particularly abdominoplasty, carry a risk of thromboembolic events due to their impact on Virchow’s triad. Vascular injury and blood stasis may result from both the surgical procedure and perioperative bed rest. Abdominoplasty ranks as the fifth most commonly requested plastic surgery worldwide, with approximately 765,248 procedures performed in 2020, according to the International Society of Aesthetic and Plastic Surgery (ISAPS).^1^^,^^2^ This procedure is commonly performed for cosmetic purposes or to address deformities following massive weight loss, particularly after bariatric surgery.^1^^,^^3^ Several risk factors associated with abdominoplasty heighten the likelihood of venous thromboembolism (VTE), including vascular trauma, general anesthesia, reduced postoperative mobility, and venous stasis in the lower extremities—especially when rectus muscle plication is performed.^4^ Deep vein thrombosis (DVT) is a major concern in some countries following abdominoplasty, with reported incidence ranging from approximately 0.3 % to 0.6 % for DVT. Pulmonary embolism (PE), although less frequent, has been documented in approximately 0.04 % of cases. In more extensive procedures (combined surgeries or circumferential abdominoplasty), the overall VTE rate may increase to 2 % or more.^5^, ^6^, ^7^ These events impose a substantial economic burden on national health insurance systems.

Pharmacological thromboprophylaxis, such as low molecular weight heparins (LMWH), effectively reduces postoperative thrombin generation.^8^^,^^9^ However, LMWH use requires careful monitoring due to potential adverse effects, including bleeding complications.^8^^,^^10^ In 2011, the American Society of Plastic Surgeons recommended the use of the Caprini Risk Assessment Model (2005 version) to stratify VTE risk and guide both mechanical and pharmacological prophylaxis in plastic surgery patients.^11^^,^^12^ In contrast, the French Society of Anesthesia and Resuscitation recommends systematic administration of LMWH for 7–10 days following abdominoplasty.^10^ Despite these recommendations, no consensus has been reached regarding the optimal VTE prevention strategy for patients undergoing abdominoplasty and many countries and centers have not yet adopted the Caprini Risk Assessment Model.^13^, ^14^, ^15^

This study retrospectively assessed the thromboembolic risk in patients who underwent abdominoplasty at a center that routinely used systematic administration of LMWH, applying the validated Caprini score for aesthetic surgery patients. We further evaluated and compared the cost-effectiveness of a Caprini score-based management strategy with that of systematic pharmacological prophylaxis at this center.

## Materials and methods

### Study characteristics

A retrospective descriptive study was conducted to review the electronic medical records of patients treated at a single academic center in France between January 2018 and July 2021. The included procedures were abdominoplasty with or without umbilical transposition and abdominoplasty combined with diastasis recti repair.

According to the center’s guidelines, all patients received LMWH (Lovenox® 4000 IU; Sanofi-Aventis, France) administered once daily, in combination with mechanical prophylaxis (compression stockings) for 15 days, regardless of their individual thromboembolic risk. In addition to pharmacological prophylaxis, all patients were prescribed thigh-high graduated compression stockings (20–30 mmHg). Patients were instructed to wear stockings continuously during hospitalization and for at least 10–14 days postoperatively, with compliance assessed at the first postoperative clinic visit.

Patients were included if they underwent one of the aforementioned procedures; those who had additional simultaneous aesthetic surgeries (e.g., breast or facial procedures) were excluded. Data extracted included patient sex, age, relevant medical history, weight loss details (e.g., body mass index, total weight loss, method of weight loss), and the components of the Caprini score (e.g., age range, type of surgery, contraceptive use, presence of sepsis). Postoperative complications such as bleeding, pulmonary embolism, and deep vein thrombosis were recorded to assess surgical outcomes.

Data collection was performed in accordance with institutional ethical guidelines, ensuring patient confidentiality.

### Statistical analysis

Data analysis was performed using Microsoft Excel 2019 and IBM SPSS Statistics version 21. A *p*-value < 0.05 was considered statistically significant. Continuous variables (age, preoperative weight loss, Caprini score, heparin duration) were reported as mean ± standard deviation and range, while categorical variables (sex, weight loss method, Caprini score category) were expressed as frequencies and percentages. Differences in mean hospital stay between patients with and without hematomas were assessed using the independent-samples *t*-test. Associations between Caprini score categories and postoperative complications were evaluated using chi-squared or Fisher’s exact tests, as appropriate. Cost-effectiveness was analyzed by comparing universal LMWH prophylaxis with Caprini-guided stratified management, calculating percentage savings and testing statistical significance. Missing data (<5 %) were addressed with complete-case analysis. Because only one VTE event occurred, no inferential comparisons between Caprini groups were performed, and these results are presented descriptively. Normality was assessed with the Shapiro–Wilk test. No corrections for multiple comparisons were applied, consistent with the exploratory nature of the study. Finally, key confounding factors such as surgeon experience, operative time, intraoperative blood loss, and postoperative protocols were not consistently available, which precluded multivariate analysis. Accordingly, our statistical findings should be interpreted as exploratory.

## Results

A total of 375 patient records were retrieved, of which 219 met the study's inclusion criteria. Four experienced senior surgeons were responsible for performing these procedures. Among the 219 patients included in this study, 200 were women and 19 were men. The mean (±standard deviation) age of patients recruited for this study was calculated to be 42.94 ± 11.7 years (range: 20–75 years). Individually calculated, the mean age of female and male patients was found to be 43.26 ± 11.8 years (range: 20–75 years) and 39.63 ± 10.1 years (range: 28–62 years), respectively. A total of 123 patients (56.16 %) were below 40 years of age. Postpartum weight loss (33.33 %), diet and sports (22.05 %), and gastric bypass (21.15 %) were the main modes of weight loss in the study cohort. The mean preoperative weight loss was 26.61 ± 23.22 kg (range: 0–100 kg). The mean duration of postoperative heparin treatment was 14.9 ± 1.5 days (range: 7–28 days). The relevant medical histories of all patients have been summarized in Table 1.

**Table 1:** Medical history of the study participants.

		Frequency (%)
Past medical history	DVT	7 (3.15)
	Sleep apnea	2 (0.9)
	Asthma	8 (3.6)
	Cerebro vascular accident	1 (0.45)
	Depression	1(0.45)
	Diabetes	1(0.45)
	Diabetes + Hypertension	8(3.6)
	Diabetes + hypothyroidism	1(0.45)
	Epilepsy	2 (0.9)
	Fibromyalgia	1(0.45)
	Hypertension	11 (4.95)
	Hypertension & Asthma	1(0.45)
	Hypertension & Hypercholesterolemia	2 (0.9)
	Hypertension & Hypothyroidism	1(0.45)
	Hypothyroidism	9 (4.05)
	Verneuil’s disease	1(0.45)
	Psoriasis	1(0.45)
	HIV	1(0.45)
Weight loss method	Gastric band	1(0.45)
	Gastric Bypass	47 (21.15)
	Gastric band + Bypass	1(0.45)
	Sleeve	35 (15.75)
	Sleeve + Bypass	1(0.45)
	Sleeve + Post partum	1(0.45)
	Lipodystrophy	1(0.45)
	Diet + Sports	49 (22.05)
	Diet + sports + Postpartum	10 (4.5)
	Post partum	73(33.33)
BMI range	18–24,9	69 (31.05)
	25–29,9	125 (56.25)
	30–34,9	22 (9.9)
	35–40	3 (1.35)
Tobacco consumption	Smoker	54 (24.6)
	Nonsmoker	153 (69.9)
	Unknown	12 (5.5)
Average Heparin treatment (days) (Mean ± SD)	Low risk	14.89 ± 0.31
	Moderate risk	14.88 ± 1.46
	High risk	15.24 ± 3.4

The mean Caprini score was 3.29 ± 1.12 (range: 1–10). The main patient characteristics related to the Caprini score have been summarized in Table 2. The most frequent 1-point risk factors were age 41–60 years and BMI > 25 kg/m², each present in 37.4 % of patients (n = 82). Varicose veins (1.8 %), pregnancy or postpartum status (0.9 %), past miscarriage (0.45 %), and hormonal contraception use (0.45 %) were rare. Among 2-point factors, all patients (100 %) underwent major open surgery lasting more than 45 min, contributing 438 points. Other 2-point factors were less common, including age 61–74 years (5.9 %), laparoscopy > 45 min (0.9 %), and cancer (0.4 %). Seven patients (3.1 %) had a personal history of deep vein thrombosis, scoring 21 points as a 3-point factor. Only one patient (0.4 %) was aged ≥75 years. Two patients (0.9 %) underwent elective arthroplasty, a 5-point factor. Overall, the total cumulative score for all patients was 650 points, largely driven by the universal presence of major open surgery and frequent age- and BMI-related factors.

**Table 2:** Model elements in the Caprini assessment among study patients.

Element of the score		N	Percentage (%)	Points
1 point	Age 41–60 years	82/219	37.4	82
	Minor surgery	0/219	0	0
	BMI > 25 Kg/m^2^	82/219	37.4	82
	Swollen legs	0/219	0	0
	Varicose veins	4/219	1.8	4
	Pregnancy or post-partum	2/219	0.9	2
	Past history of miscarriage	1/219	0.45	1
	Hormonal contraception	1/219	0.45	1
	Sepsis < 1 month	0/219	0	0
	Severe pulmonary disease	0/219	0	0
	Abnormal lung function	0/219	0	0
	Acute myocardial infarction	0/219	0	0
	Congestive heart failure < 1 mois	0/219	0	0
	Past history of inflammatory bowel disease	0/219	0	0
	Non surgical bed rest	0/219	0	0
2 points	Age 61–74 years	13/219	5.9	
	Arthroscopy	0/219	0	0
	Major open surgery > 45 min	219/219	100	438
	Laparoscopy > 45 min	2/219	0.9	4
	Cancer	1/219	0.4	2
	Bed rest > 72 h	0/219	0	0
	Immobilization with plaster	0/219	0	0
	Central venous catheter	0/219	0	0
3 points	Age ≥ 75 ans	1/219	0.4	3
	Personal history of DVT	7/219	3.1	21
	Family history of DVT	0/219	0	0
	Mutation de Leiden’s factor V	0/219	0	0
	Mutation 20210A of prothrombin	0/219	0	0
	Lupus Anticoagulants	0/219	0	0
	Anti cardiolipin Antibodies	0/219	0	0
	Hyperhomocysteinemia	0/219	0	0
	Heparin induced Thrombocytopenia	0/219	0	0
5 points	Cerebrovascular accident < 1 month	0/219	0	0
	Elective Arthroplasty	2/219	0.9	10
	Pelvis, hip or leg fracture	0/219	0	0
	Acute spinal lesion < 1 month	0/219	0	0
Total points				**650**

**Figure 1:**
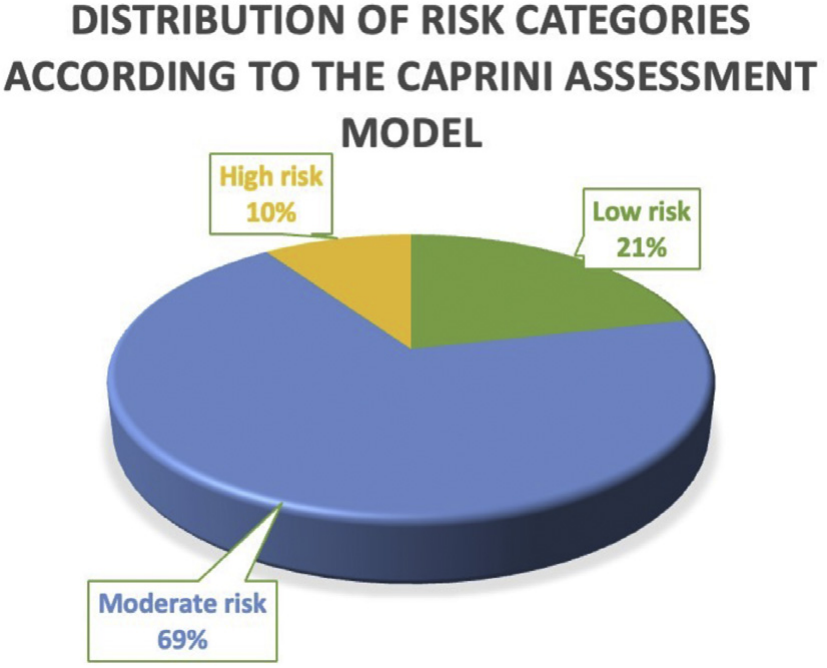
Distribution of patients by risk category according to the Caprini assessment model.

Most patients (69 %) were classified as having a moderate risk of thromboembolic events, while 21 % were categorized as low risk and 10 % as high risk. This distribution highlights that the majority of the study population presented a moderate risk profile, with a smaller proportion at high risk, which has implications for perioperative thromboprophylaxis strategies. The distribution of risk categories has been presented in Figure 1.

Table 3 summarizes the characteristics of patients who experienced postoperative complications. The majority of complications were hematomas, with one case (0.45 %) of pulmonary embolism occurring in a 47-year-old overweight woman who was post-partum, underwent abdominoplasty with umbilical transposition surgery, and had a moderate Caprini score of 4 (moderate risk). Patients with hematomas (5.93 %) were predominantly female (12 out of 13 cases) and had diverse comorbidities such as smoking, hypertension, diabetes, or asthma. The Caprini scores ranged from 2 to 4, corresponding to low or moderate thromboembolic risk. Most patients underwent abdominoplasty with umbilical transposition, and rectus muscle diastasis closure was performed in three cases. Weight loss methods included bariatric surgery (bypass or sleeve), diet and sports, or were unknown. Heparin therapy duration was consistently 15 days, while hospital stays varied from 2 to 9 days. These findings suggest that postoperative hematomas were relatively common regardless of individual risk factors.

**Table 3:** Characteristics of the patients showing postoperative complications.

Complication	Age	Sex	Past history	Type of surgery	Method of weight loss	Caprini score	Risk level	Duration heparin therapy	Duration hospitalization
Pulmonary embolism	47	F	Non-smoker	ADLTU	Post-partum	4	Moderate	15	1
Hematoma	36	F	Smoker, hypertension	ADLTU + RD	Post-partum	2	Low	15	6
Hematoma	28	F	Non-smoker	ADLTU	Bypass	2	Low	14	7
Hematoma	53	F	Non-smoker	ADLTU + RD	Unknown	4	Moderate	15	4
Hematoma	51	F	Smoker	ADLTU + RD	Bypass	3	Moderate	15	5
Hematoma	41	F	Non-smoker	ADLTU	Diet + sports	3	Moderate	15	6
Hematoma	56	F	Diabetes	ADLTU	Unknown	4	Moderate	15	3
Hematoma	35	M	Non-smoker	ADLTU	Bypass	2	Low	15	4
Hematoma	51	F	Hypertension, Asthma	ADLTU	Diet + sports	4	Moderate	15	4
Hematoma	37	F	Non-smoker	ADLTU	Unknown	3	Moderate	15	2
Hematoma	62	F	Hypertension, Diabetes	ADLTU	Sleeve	3	Moderate	15	4
Hematoma	33	H		ADLTU	Sleeve	4	Moderate	15	9
Hematoma	51	F		ADLTU	Bypass	3	Moderate	15	6
Hematoma	42	F	Asthma, smoker	ADLTU + RD	Diet + sports	3	Moderate	15	5

ADLTU: Abdominal with transposition of umbilicus.

ADLTU + RD: Abdominal with transposition of umbilicus + and rectus muscle diastasis closure.

Table 4 compares demographic and clinical risk factors between patients who developed postoperative hematomas and those who did not. The mean age was similar in both groups (44.3 ± 10.3 years vs. 42.9 ± 11.8 years, *p* = 0.64), as was sex distribution (*p* = 0.31). However, the duration of hospitalization was significantly longer for patients with hematomas (5 ± 1.8 days) compared to those without (3.7 ± 2.1 days, *p* = 0.02). The duration of heparin treatment, BMI distribution, Caprini risk level, and mean Caprini score showed no significant differences between the two groups (all *p* > 0.05). These findings suggest that while most baseline characteristics were comparable, postoperative hematomas were associated with a prolonged hospital stay.

**Table 4:** Risk factors for hematoma among study patients.

Risk factors	Patients with hematoma (*n* = 13)	Patients without hematoma (*n* = 206)	*P*-value
Age (years)	44.31 ± 10.32	42.85 ± 11.8	0.643
Sex	M: 2 (15.4 %)	M: 17 (8.3 %)	0.317^b^
	F: 11 (84.6 %)	F: 189 (91.7 %)	
Duration hospitalization(days)	5 ± 1.8	3.74 (±2.07)	0.020*
Duration heparin treatment	14.92 ± 0.27	14.92 (±1.6)	0.711
BMI range	<25 kg/m^2^: 5 (38.5 %)	<25 kg/m^2^: 77 (37.4 %)	0.570^a^
	≥25 kg/m^2^: 8 (61.5 %)	≥25 kg/m^2^: 129 (62.6 %)	
Risk level	Low: 3 (23.1 %)	Low: 44 (23.8 %)	0.732
	Moderate: 10 (76.9 %)	Moderate: 141 (76.2 %)	
Mean Caprini score	High: 0	High: 21	0.983
	3.07 ± 0.75	3.31 ± 1.14	

P: Independent samples *t*-test.

a Pearson X2 test.

b Exact probability test.

⁎ *P* < 0.05 (significant).

**Table 5:** Comparison of costs of the French thromboprophylaxis strategy to the Caprini model-derived management. The Caprini score was utilized to stratify patients in the study, offering a perspective that using this scoring system to guide treatment plans could lead to cost variations compared to the standard administration of heparin therapy to the 219 patients from the Hospital of a French university center. It is noted that the costs and tariffs presented in the study were extrapolated from ameli.fr and Vidal.fr at the time of publication, indicating that the financial implications of heparin therapy were based on data from these sources.

STRATEGY		Cost						
				Check up				Total
Practice at a French University Center using: Systematic heparin therapy During 14.9 days from the of hospital discharge		*Lovenox 0.4: 1 vial SC/ 24 h*	*Daily injection by nurse*	*Blood sample collection once a week*	*Physician consultation*	*Platelet count once a week*	*Compressive Stocking (1 pair per patient)*	53,015, 49 €
		3267 days x 8.97 = 29,304.99 €	3267 days x 4,5 € = 14,701.5 €	466 (i.e., 3267/7) weeks x 4.73 € = 2204.18 €	466 × 25€ = 11,650 €	466 × 4 € = 1864 €	219 × 29.78 = 6521.82 €	
Stratification based on Caprini Score	Low risk *47 patients*	No heparin = 0 €	NA	NA	NA	NA	47 × 29.78 = 1399.66 €	1399.66 €
	Moderate risk^a^ *151 patients*	Lovenox 0.4: 1 vial SC/ 24h1 057 days x 8,97 € = 9481,29 €	1057 × 4.5 € = 4756.5 €	151 weeks x 4.73 € = 714.23 €	151 × 25 € = 3775 €	151 × 4 € = 604 €	151 × 29.78 € = 4496.78 €	23,827.8 €
	High Risk^b^ *32 patients*	Lovenox 0.4: 1 vial SC/ 24h 672 days x 8.97 € = 6027.84 €	672 × 4.5 € = 3024 €	96 Week x 4.73 € = 454.08 €	96 Week x 25€ = 2 400 €	96 × 4€ = 384 €	32 × 29.78 € = ` 952.96 €	13,242.88 €

a 151 patients x 7 days of heparin treatment recommended = 1057 days.

b 21 patients x 32 days of heparin treatment recommended = 672 days.

We compared the total costs of two postoperative prophylaxis strategies after abdominoplasty in this large cohort (Table 5). The first strategy consisted of systematic heparin therapy for all patients, as implemented in our case series, resulted in a total cost of approximately €53,015.49. This includes heparin injections, nursing care, weekly blood tests, physician consultations, platelet counts, and compressive stockings. In contrast, a risk-stratified approach based on the Caprini score significantly reduced costs by tailoring prophylaxis to individual risk levels. For low-risk patients (*n* = 47), no heparin was used, with costs limited to compressive stockings (€1399.66). Moderate-risk patients (*n* = 151) received 7 days of heparin therapy, leading to a total cost of €23,827.8. High-risk patients (*n* = 32) required extended heparin therapy for 32 days, generating a cost of €13,242.88. This comparison demonstrates that Caprini score-based stratification can markedly reduce overall costs while adapting prophylaxis to patient risk profiles.

## Discussion

This retrospective study evaluated venous thromboembolism prevention strategies in 219 patients undergoing abdominoplasty in a French center where systematic 15-day postoperative LMWH prophylaxis was routinely used. We retrospectively applied the validated 2005 Caprini Risk Assessment Model to this cohort and compared the current practice with a Caprini-based strategy. Our cost analysis showed that adopting a risk-stratified approach could reduce LMWH use by 44.5 %, translating into significant annual savings for the French healthcare system while avoiding unnecessary anticoagulant exposure.

Abdominoplasty carries one of the highest thromboembolic risks among aesthetic procedures, mainly due to venous stasis, rectus plication, and postoperative immobility.^16^ Pulmonary embolism remains a feared complication, with reported incidence rates ranging from 0.1 % to 1 %.^17^ In our cohort, one case of pulmonary embolism (0.45 %) occurred in a moderate-risk patient (Caprini score = 4), illustrating that thromboembolic events can still occur in patients outside the highest risk categories. Thirteen patients (5.9 %) developed postoperative hematomas, a complication potentially exacerbated by systematic LMWH use, as suggested in previous reports.^18^, ^19^, ^20^

Current practice in many countries, including France, relies on universal prophylaxis, driven partly by medicolegal considerations.^21^, ^22^, ^23^ However, several studies have demonstrated that pharmacologic prophylaxis provides the greatest benefit in high-risk patients (Caprini ≥ 7–8),^24^^,^^25^ whereas for low- and moderate-risk patients, mechanical measures and early mobilization are often sufficient.^26^^,^^27^ Our findings reinforce this evidence by showing that a large proportion of abdominoplasty patients fall into low- or moderate-risk categories (90 % in our cohort) and would likely not require systematic anticoagulation.

The Caprini score remains the most widely validated tool for VTE risk stratification in plastic surgery.^11^^,^^28^^,^^29^ Its systematic use could help avoid unnecessary anticoagulation, thereby reducing complications such as hematoma, prolonged hospitalization, and increased costs. For countries and centers that have not adopted this model, our results illustrate both the economic and clinical benefits of a risk-adapted strategy. Applying such an approach at a national level could save millions of euros annually, as estimated from ISAPS data.^30^

Nevertheless, the Caprini model is not without limitations.^31^ It was developed heuristically and may overestimate risk, with up to 97 % of patients identified as “at risk” never developing VTE. Moreover, several authors have noted that most postoperative DVTs occur in patients with intermediate scores (2–8),^14^^,^^16^ highlighting the need to consider additional procedure-specific factors not captured by the score. Therefore, while the Caprini score is a valuable framework, it should be combined with clinical judgment and procedural considerations to guide decision-making.

Beyond the limitations of the Caprini score itself, our study also carries methodological constraints. The cohort was not determined by an a priori power calculation, and the low incidence of VTE limited our ability to detect risk group differences. Important confounders such as surgeon experience, operative time, intraoperative blood loss, surgical technique, and postoperative care protocols were not consistently available, preventing multivariate analysis. The cost analysis was based on fixed institutional assumptions without sensitivity testing, so variations in drug pricing, complication costs, or patient adherence could substantially change the economic conclusions. Compliance with mechanical prophylaxis, although prescribed, was not systematically documented, reducing reproducibility. Finally, the retrospective nature of data collection meant that some baseline clinical and operative characteristics were incompletely captured. Taken together, these factors highlight the exploratory nature of our findings and underscore the need for prospective, multicenter studies with standardized data collection to validate the role of Caprini-guided prophylaxis in abdominoplasty.

## Conclusions

Adopting a Caprini-based risk-stratified approach could reduce low molecular weight heparins use and healthcare costs without compromising patient safety. For centers and countries that do not routinely use the Caprini score, its implementation represents an opportunity to avoid unnecessary anticoagulation and associated complications while promoting more rational and sustainable perioperative care.

## Funding

This research received no external funding.

## Author contributions

Conceptualization, A.A. and A.S.; methodology, A.A., E.L.; investigation, A.A., A.S. and D.G.; data curation, S.G., E.L.; writing—original draft preparation, A.A., A.S., and D.G.; Resources: D.G., S.G., H.M.; writing—review and editing, H.M., S.G., E.L. All authors have read and agreed to the published version of the manuscript.

## Ethical approval

The study was conducted in accordance with the Declaration of Helsinki and approved by the Institutional Review Board of University Institute for Locomotion and Sports (protocol code [*IRB00014528_2025_35*] and date of approval [25/07/2025]).

## Informed consent statement

Patient consent was waived due to the retrospective nature of the study and the use of anonymized data extracted from existing medical records, in accordance with institutional policies.

## Declaration of competing interest

The authors declare no conflicts of interest.
