# Corrigendum to “Utility of the caprini risk assessment model in guiding venous thromboembolism prophylaxis after abdominoplasty” [JPRAS Open 46 (2025) 305–315]

**DOI:** 10.1016/j.jpra.2026.01.001

**Published:** 2026-01-12

**Authors:** Abdulaziz Asiry, Anas Sayegh, Dimitri Gangloff, Hatan Mortada, Silvia Gandolfi, Elise Lupon

**Affiliations:** a College of Medicine, King Saud Bin Abdulaziz University for Health Sciences, Jeddah, Saudi Arabia; b King Abdullah International Medical Research Center, Jeddah, Saudi Arabia; c Department of Surgery, Ministry of the National Guard–Health Affairs, Jeddah, Saudi Arabia; d Department of Surgery, Faculty of Medicine, Jazan University, Jazan, Saudi Arabia; e Plastic and Reconstructive Surgery Department, Rangueil University Hospital Center of Toulouse, Toulouse, France; f Department of Plastic Surgery and Burn Unit, King Saud Medical City, Riyadh, Saudi Arabia; g Plastic and Reconstructive Surgery Department, Institut Universitaire Locomoteur et du Sport, Pasteur 2 Hospital, Nice, France; h Université Côte d'Azur, CNRS, LP2M, Nice, France

The authors regret that the affiliations of some co-authors were not correctly listed in the original publication. The corrected affiliations are provided above.

The authors would like to apologize for any inconvenience caused.

